# The Brain Mechanisms of Music Stimulation, Motor Observation, and Motor Imagination in Virtual Reality Techniques: A Functional Near-Infrared Spectroscopy Study

**DOI:** 10.1523/ENEURO.0557-24.2025

**Published:** 2025-06-27

**Authors:** Junjie Liang, Boyuan Liang, Zengquan Tang, Xingchen Huang, Sitong Ou, Chunli Chang, Yujue Wang, Zishu Yuan

**Affiliations:** ^1^Department of Rehabilitation, The Fifth Affiliated Hospital of Guangzhou Medical University, Guangzhou 510700, China; ^2^The Guangzhou Medical University, Guangzhou 511436, China

**Keywords:** action observation, functional near-infrared spectroscopy (fNIRS), motor imagination, music stimulation, virtual reality

## Abstract

Virtual reality (VR) has gained popularity in recent years, integrating with conventional music stimulation (MS), action observation (AO), and motor imagination (MI). It offers promising opportunities for developing innovative rehabilitation treatments, though the mechanisms underlying these effects remain unclear. This study aims to compare brain activation and network mechanisms following the fusion of MS, AO, and MI with VR. Fifty healthy participants were recruited and underwent functional near-infrared spectroscopy synchronization with three VR tasks: MS (VRMS), AO (VRAO), and MI (VRMI). The results indicate that VRMS significantly enhances functional connectivity of the bilateral primary sensory cortex (S1), premotor cortex, and supplementary motor area (PM&SMA) compared with VRAO and VRMI. Furthermore, the interaction among the bilateral PM&SMA, right dorsolateral prefrontal cortex, and right primary motor cortex (M1) regions is notably stronger with VRMS than with the other VR tasks. These findings elucidate the brain activation and network characteristics of the three VR tasks, highlighting VRMS's potential in boosting the functional interaction among brain regions. Future research should explore additional brain regions, broader diseased brain samples, and alternative brain-stimulation effects of VRMS.

## Significance Statement

As established noninvasive neuromodulation modalities, music stimulation (MS), action observation (AO), and motor imagery (MI) face two clinical constraints: overly simplistic task design reducing therapeutic salience and compromised patient engagement. Virtual reality (VR) integration addresses these limitations through enhanced immersive and multisensory stimulation. This study investigates cortical activation dynamics and functional network during VRMS, VRAO, and VRMI tasks. The functional near-infrared spectroscopy results demonstrate that VRMS induces superior functional connectivity modulation compared with VRAO and VRMI tasks, particularly within sensorimotor integration. These finding may contribute to optimizing VR rehabilitation paradigm selection and advancing VR technology exploration.

## Introduction

With aging populations and an increasing incidence of neurological diseases, there is a growing demand for functional recovery and rehabilitation, prompting research into new methods (Stinear et al., 2020; O'Dell, 2023). Music stimulation (MS), action observation (AO), and motor imagination (MI) are noninvasive rehabilitation techniques widely used in clinical practice, especially in community and family therapy settings. These techniques achieve significant therapeutic outcomes by using sound and vision to activate the cognitive and motor cortices. Moreover, virtual reality (VR) technology, as an emerging advanced tool, has the potential to address the limitations of traditional rehabilitation methods, such as monotony and patient focus challenges. With the integration of VR, these techniques can address these issues, allowing professionals to manage multiple patients more efficiently and consequently reduce their workload. These rehabilitation methods require minimal motor ability from patients and can be easily administered in a seated position. When combined with VR, they create an immersive and multisensory stimulation without increasing the patients’ risk of falls (Laver et al., 2017; Palma et al., 2017).

Musical stimuli utilize elements such as tone, rhythm, and melody to activate specific brain areas, thereby increasing cortical activation in auditory and motor regions. This mechanism facilitates brain-stimulation treatment (Sihvonen et al., 2017; Buard et al., 2019; de Witte et al., 2020; Chen et al., 2022). Research demonstrates that music can stimulate bilateral brain networks in subcortical regions, including the frontal, parietal, and temporal lobes, which promotes neuroplasticity. Moreover, music may enhance cognitive function, alleviate symptoms of anxiety and depression, and potentially improve cognitive abilities (Chorna et al., 2019; Salihu et al., 2024).

AO involves perceiving and understanding motor features by watching others’ movements through videos or other media, leading to passive imitation and psychological-level movement execution (Wulf et al., 2010). This process may activate the brain's mirror neuron system (MNS) and various regions associated with visual processing, including the occipital, temporal, and parietal areas, as well as specific parts of the inferior parietal lobe and frontal gyrus. Motor imagery (MI), building on AO, requires autonomously imagining motor actions, actively inducing psychological imitation, and executing movements, thereby activating the MNS. Both AO and MI involve internal mental activities that do not result in physical motor output but stimulate the motor cortex similarly to actual movement execution (Hardwick et al., 2018; Bruton et al., 2020).

The prefrontal cortex (PFC) is a crucial brain region responsible for coordinating higher cognitive functions, including memory, language, and executive functioning (Siddiqui et al., 2008). The PFC is involved in music therapy, movement observation, and MI. For instance, music therapy facilitates the integration and coordination of cognitive functions such as attention, imagination, creativity, memory, and emotion through neural plasticity (Speranza et al., 2022). When individuals engage in motor observation and MI, the primary motor area (M1), premotor cortex (PM), and supplementary motor area (SMA) of the frontal lobe are activated (Larsson et al., 1996; Bordoloi et al., 2024). Thus, during music therapy, the unconscious processes of beat perception and prediction, as well as the observation and imagination of regular movement, are closely linked to M1, PM, and SMA. The prefrontal cognitive area and motor area are essential in regulating music therapy, motor observation, and MI.

These techniques are frequently combined in clinical settings to enhance motor relearning and adaptation (Rannaud Monany et al., 2022). Despite the effectiveness of these rehabilitation technologies, several technical shortcomings remain. These issues include reliance on a single training mode, challenges in maintaining patient attention, and obstacles to the popularization of technology, all of which hinder their efficacy and widespread adoption.

VR can be employed as a presentation technique alongside conventional rehabilitation methods. With the advancement of VR technology, its integration into daily life, architecture, military training, entertainment, medical research, and other fields has become increasingly common, establishing it as a prominent approach for rehabilitation treatment (Tieri et al., 2018). In the medical realm, Ewa Szczepocka et al. explored the integration of VR with cognitive training, discovering that it could enhance the sustained attention of the elderly, thereby suggesting the potential of VR in the cognitive domain. Their research indicates that improvements in cognitive function are closely linked to neuronal plasticity (Grealy et al., 1999; Szczepocka et al., 2024). Compared with real stimulation, VR stimulation produces a greater effect on neuroplasticity, evidenced by increased β-band power in the frontal region (Gangemi et al., 2023).

The integration of MS, AO, and MI within VR technology shows promise for (1) enhancing stimulation that targets the MNS and (2) promoting the functional integration of the MNS with sensorimotor cortical networks. Furthermore, these innovations have the potential to overcome technological limitations and their adoption challenges, thereby facilitating applications within communities and households (Merians et al., 2002; Kwakkel et al., 2004; Tieri et al., 2018).

Recently, functional near-infrared spectroscopy (fNIRS) has rapidly emerged as a powerful tool for detecting brain activity. It operates based on the interaction between near-infrared (NIR) light and the human tissue, enabling the real-time monitoring of hemodynamics in the cerebral cortex. NIR light penetrates biological tissues, such as lipids and hemoglobin, which exhibit distinct absorption characteristics at different wavelengths (Pinti et al., 2020). The most critical absorption chromophore is hemoglobin, whose physiological state can be indicated by oxygenated hemoglobin (HBO), deoxyhemoglobin, and total hemoglobin (HBT). In this study, HBO and HBT were used as analysis indices. When cognitive functions are engaged, blood flow in the PFC increases. Both HBO and HBT are sensitive to changes in cerebral blood flow and possess a good signal-to-noise ratio (Yan et al., 2024). Due to its advantages, including portability, exercise tolerance, and safety, fNIRS has been widely adopted in neurological research.

In our study, we examined the effects of three tasks on brain activation and brain networks. fNIRS perfectly aligns with our focus on monitoring cortical effects while simplifying our experimental procedures because of its portability and safety. However, additional information about the effects and mechanisms of brain stimulation following the combination of VR with these three rehabilitation technologies is needed, especially regarding the differences in their effect sizes. This study, therefore, explored the effects of integrating VR with MS (VRMS), AO (VRAO), and MI (VRMI) on brain activation and networks. This research also provides a theoretical basis for clinical application.

## Materials and Methods

### Participants

Fifty healthy participants were recruited for the study conducted at the Fifth Affiliated Hospital of Guangzhou Medical University from July 2023 to March 2025. The participants had an average age of 20.3 ± 0.8 years and comprised 20 males and 30 females. None had cognitive or intellectual disabilities, had undergone VRMS, or received formal training in music. All participants provided written informed consent. The study was approved by the Ethics Committee of Guangzhou Medical University (GYWY-L2023-58) and registered with the International Clinical Trials Registry (ChiCTR2300071527).

### Experiment

Before the experiment, the testing environment was maintained in silence, and lighting was controlled to minimize auditory and visual interference during fNIRS data collection. Participants used Huawei (China) head-mounted displays, with brightness adjusted for clear visibility of the experimental stimuli. They sat comfortably in a chair with a backrest while wearing the fNIRS equipment and VR glasses. A researcher briefed them on the study protocol, emphasizing the importance of focusing on the task and refraining from talking during the session. Preliminary fNIRS data were collected to ensure a stable channel signal.

**Figure 1.**
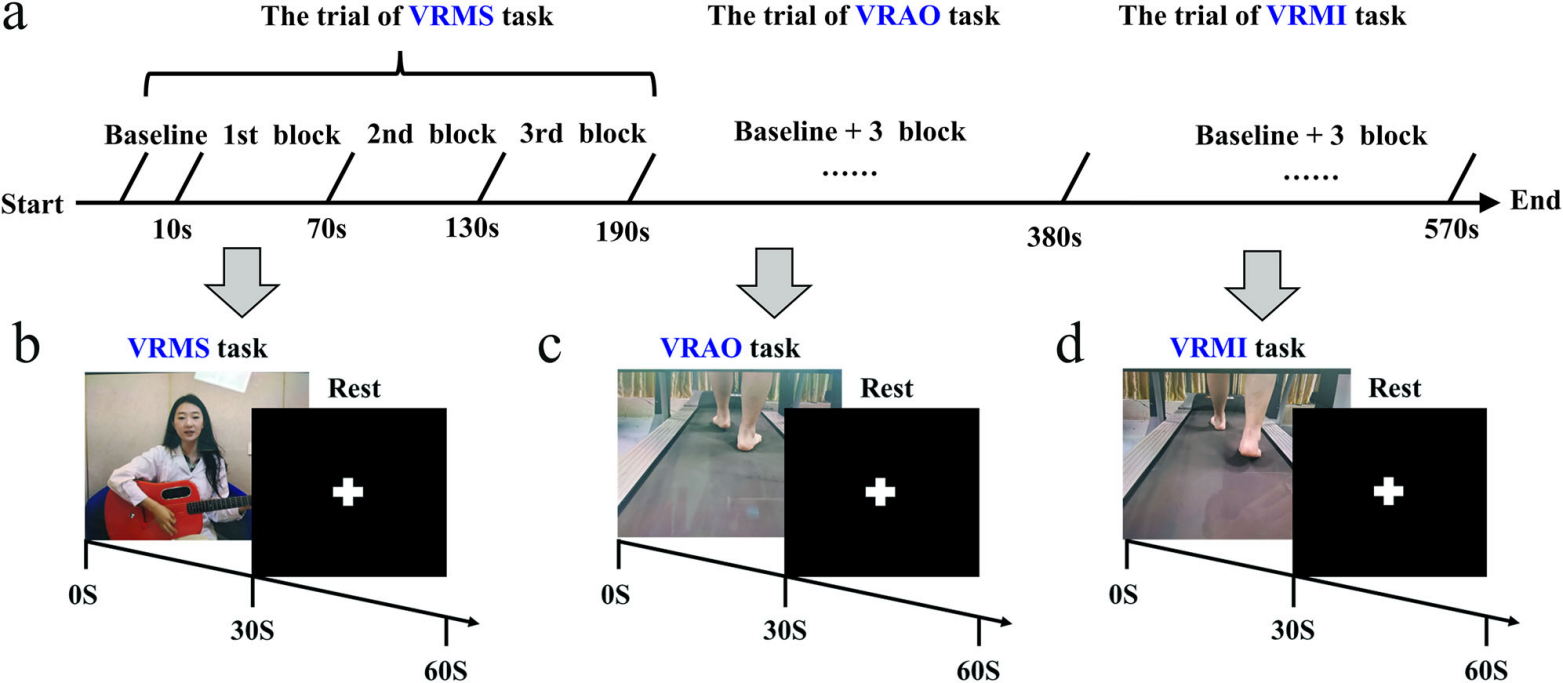
Experimental design flowchart. ***a***, Overview of the experimental procedure; (***b***) VRMS block; (***c***) VRAO block; (***d***) VRMI block. VRMS, virtual reality music stimulation; VRAO, virtual reality action observation; VRMI, virtual reality motor imagination.

The study used fNIRS to synchronize VRMS, VRAO, and VRMI tasks within a block design format. Each task consisted of three blocks that were identical for all participants. Before each block, participants had 10 s to prepare. Each block included a 30 s task period followed by a 30 s recovery period, during which participants viewed a white crisscross pattern on a black screen. There were no breaks between consecutive blocks or tasks. Baseline data were collected for 10 s before the onset of each block, resulting in a total trial duration of 190 s (Fig. 1).

In the VRMS task, participants listened to the first verse of the song “Red River Valley” in Mandarin. The piece was performed in 4/4 time, in the key of F major, at a tempo of 120 bpm, and consisted of 90 notes in total. The VRAO task involved observing a video of a walk from a third-person perspective, requiring sustained, focused attention. Similarly, the VRMI task featured the same walking video from a third-person perspective but included synchronized auditory cues designed to evoke kinesthetic imagery of walking in the participants. Throughout the experiment, we collected hemodynamic data from specified brain regions of interest (ROIs) using fNIRS.

### fNIRS settings

**Figure 2.**
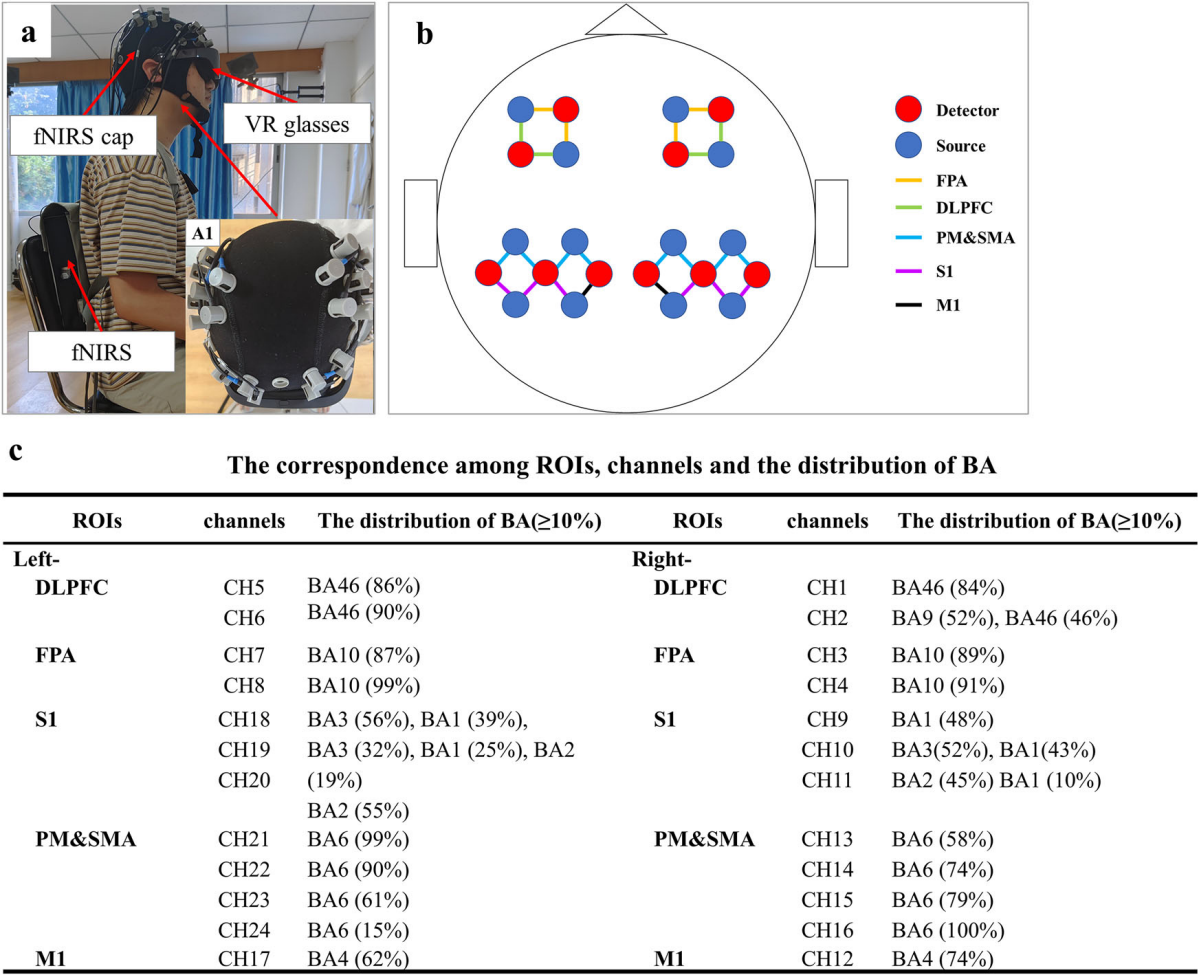
Brain region locations of specific fNIRS channels. ***a***, The subject's posture during the experiment. ***A*1**, Schematic diagram showing the fNIRS device positioned on the subject's head. ***b***, Schematic of the light sources, detectors, and fNIRS channels; different colors indicate the distribution across brain regions. ***c***, Distribution of ROIs corresponding to each channel in the FASTRAK 3D digital positioning system, based on BA standards (Amunts and Zilles, 2015). DLPFC, dorsolateral prefrontal cortex; FPA, frontopolar area; S1, primary sensory cortex; PM&SMA, premotor cortex and supplementary motor area.

Data were collected using fNIRS technology (Nirsmart, Danyang Huichuang Medical Equipment), continuously measuring changes in oxyhemoglobin and methemoglobin within the cerebral cortex. This was achieved using 760 and 850 nm light wavelengths. The sampling frequency was set at 11 Hz. The recordings utilized 24 channels, incorporating 10 detectors and 12 light sources, with each detector positioned 30 mm from its corresponding light source. This configuration aligns with the international 10–20 standard for lead-system design (Fig. 2*a*).

The ROIs identified in this study were the right and left dorsolateral PFC (RDLPFC and LDLPFC), the right and left front polar area (RFPA and LFPA), the right and left primary sensory cortex (RS1 and LS1), the right and left PM (RPM and LPM), the supplementary motor cortex (SMA), and the right and left primary motor cortex (RM1 and LM1, respectively). A FASTRAK three-dimensional (3D) digitization instrument (Polhemus, Colchester) was used to position the ROIs on the brain. The instrument sequentially tracked the 24 channels in the fNIRS, collecting data on the *X*, *Y*, and *Z* axes of each light source and detector with reference to Brodmann areas (BA) standards (Fig. 2*b*).

Each ROI was mapped to a distinct BA. Channel assignments to ROIs were determined by identifying the BA subregion with maximum spatial overlap at each channel location. Channels colocalized within an ROI collectively delineated the target region (Fig. 2*c*).

### Signal processing

The fNIRS data collected were imported into the NirSpark software (Danyang Huichuang Medical Equipment) for preprocessing. Prominent motion artifacts were manually corrected, and a bandpass filter (0.01–0.2 Hz) was applied to remove residual motion artifacts and physiological noise (de Witte et al., 2020; Sutoko et al., 2020). This study investigates the activation effects induced by VR tasks, particularly focusing on the task-related response values of HBO and HBT. The light signal was converted into HBO and HBT according to the Lambert–Beer law, and the average signal for ROIs within each block was calculated.

In the brain-network module, functional connectivity (FC) values quantify the connections both between channels and between ROIs. Homologous brain-network FC values assess the relationships among channels within the same ROI, whereas heterologous brain-network FC values gauge the overall connection strength between different ROIs. To ensure the reliability of the statistical results, FC values were corrected for multiple comparisons across ROIs using the false discovery rate (FDR) method.

### Statistical analysis

The data were analyzed using the Kolmogorov–Smirnov normality test via SPSS version 22.0. For data adhering to a normal distribution, paired-sample *t* tests were conducted. Conversely, the nonparametric Wilcoxon signed-rank test was employed for two related samples that did not satisfy normality assumptions. A significance threshold of *p* < 0.05 was set for statistical interpretation. Image processing was carried out using the NirSpark software and GraphPad 9.0.

## Results

**Figure 3.**
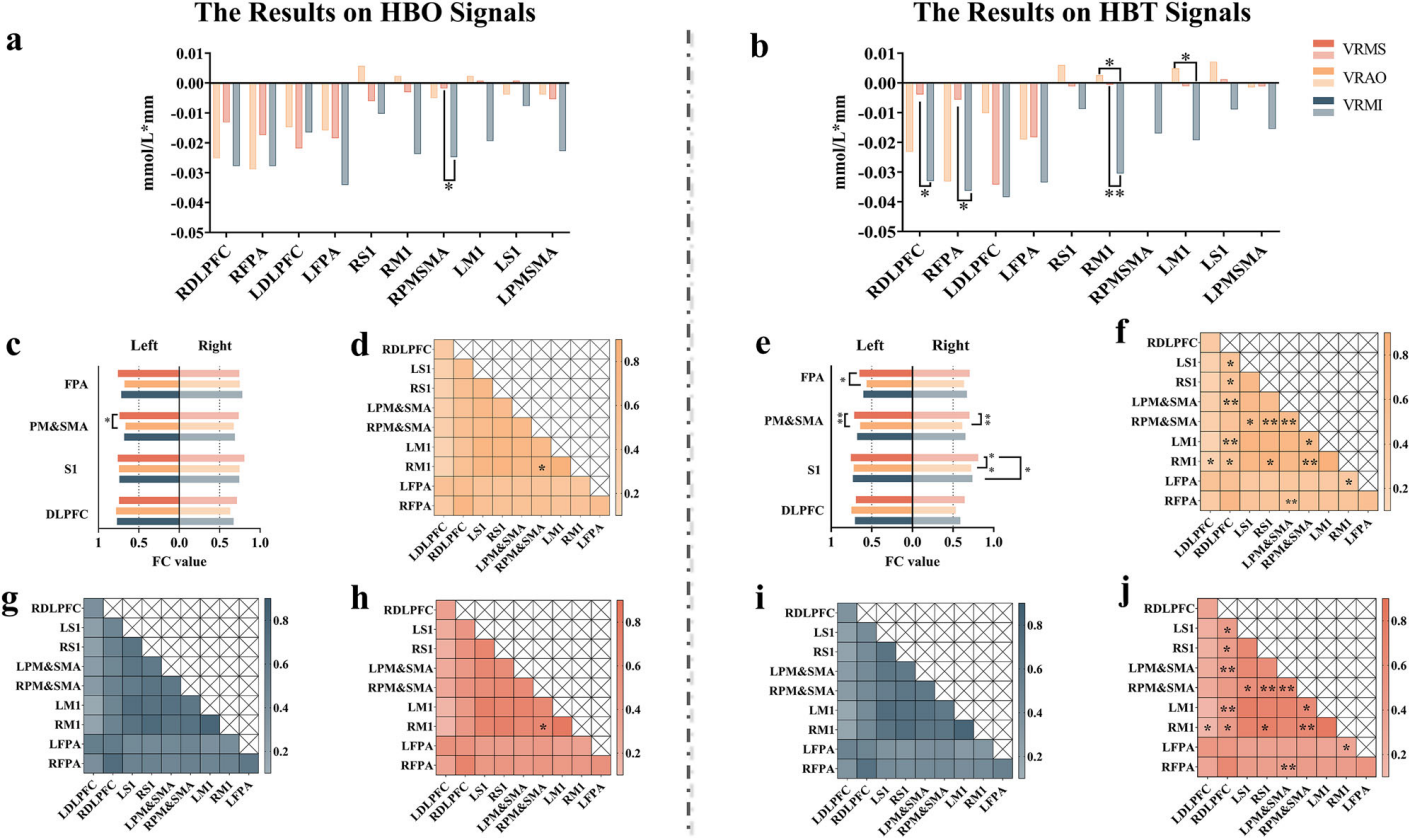
Brain activation and network connectivity results. VRMS, virtual reality music stimulation; VRAO, virtual reality action observation; VRMI, virtual reality motor imagination; HBO, oxygenated hemoglobin; ROIs, regions of interest. ***a***, ***b***, Brain activation maps for HBO and HBT, respectively. ***c***, ***e***, Homologous brain networks based on HBO and HBT, respectively. ***d***, ***h***, ***g***, Heterogeneous brain networks (HBO) for VRMS, VRAO, and VRMI, respectively. ***f***, ***j***, ***i***, Heterogeneous brain networks (HBT) for VRMS. Significance levels: *p < 0.05; **p < 0.01. All the significant differences based on FDR correction.

In the comparison of ROIs activation on HBO, VRAO exhibited a higher activation degree in the right premotor supplementary motor area (RPMSMA) than VRMI (*p* < 0.05; Fig. 3*a*; Table 1). Regarding ROI activation on HBT, VRMS displayed significantly increased activation in both RM1 and LM1 compared with VRMI (*p* < 0.05; Fig. 3*b*; Table 2). Additionally, VRAO demonstrated significantly increased activation in RDLPFC and RFPA (*p* < 0.05), as well as in RM1 (*p* < 0.01; Fig. 3*b*; Table 2).

**Table 1.** Comparison of the task effect values of HBO in the different ROIs

ROIs	Mean ± standard deviation			VRMS vs VRAO		VRMS vs VRMI		VRAO vs VRMI	
	VRMS	VRAO	VRMI	*t* value	*p* value	*t* value	*p* value	*t* value	*p* value
RDLPFC	−0.025 ± 0.063	−0.013 ± 0.06	−0.028 ± 0.073	−0.995	0.325	0.193	0.848	1.032	0.307
RFPA	−0.029 ± 0.087	−0.017 ± 0.07	−0.028 ± 0.073	−0.705	0.484	−0.078	0.938	0.685	0.497
LDLPFC	−0.015 ± 0.06	−0.022 ± 0.083	−0.017 ± 0.121	0.523	0.603	0.099	0.921	−0.272	0.787
LFPA	−0.016 ± 0.063	−0.019 ± 0.062	−0.034 ± 0.084	0.207	0.837	1.245	0.219	1.036	0.305
RS1	0.006 ± 0.047	−0.006 ± 0.05	−0.01 ± 0.056	1.395	0.169	1.680	0.099	0.471	0.640
RM1	0.002 ± 0.06	−0.003 ± 0.051	−0.024 ± 0.063	0.455	0.651	2.000	0.051	1.984	0.053
RPMSMA	−0.005 ± 0.04	−0.002 ± 0.048	−0.025 ± 0.06	−0.351	0.727	1.896	0.064	2.537	0.014*
LM1	0.002 ± 0.057	0.001 ± 0.048	−0.019 ± 0.072	0.148	0.883	1.681	0.099	1.733	0.089
LS1	−0.004 ± 0.044	0 ± 0.045	−0.008 ± 0.058	−0.554	0.582	0.406	0.687	0.945	0.349
LPMSMA	−0.004 ± 0.047	−0.005 ± 0.047	−0.023 ± 0.06	0.156	0.876	1.689	0.098	1.903	0.063

“L” represents the left side; “R” represents the right side. VRMS, virtual reality music stimulation; VRAO, virtual reality action observation; VRMI, virtual reality motor imagination; HBO, oxygenated hemoglobin; ROIs, brain regions of interest.

* *p* < 0.05.

**Table 2.** Comparison of the task effect values of HBT in the different ROIs

ROIs	Mean ± standard deviation			VRMS vs VRAO		VRMS vs VRMI		VRAO vs VRMI	
	VRMS	VRAO	VRMI	*t*/*z* value	*p* value	*t*/*z* value	*p* value	*t* value	*p* value
RDLPFC	−0.023 ± 0.058	−0.004 ± 0.074	−0.033 ± 0.080	−1.453^a^	0.146	0.746	0.459	2.129	0.038*
RFPA	−0.033 ± 0.084	−0.006 ± 0.073	−0.036 ± 0.079	−1.989	0.052	0.220	0.827	2.297	0.026*
LDLPFC	−0.010 ± 0.061	−0.034 ± 0.089	−0.038 ± 0.128	−0.864^a^	0.388	1.440	0.156	0.242	0.810
LFPA	−0.019 ± 0.064	−0.018 ± 0.055	−0.034 ± 0.082	−0.061	0.952	−0.738^a^	0.460	1.136	0.261
RS1	0.006 ± 0.045	−0.001 ± 0.045	−0.009 ± 0.065	−0.265^a^	0.791	1.428	0.160	0.774	0.442
RM1	0.003 ± 0.054	−0.001 ± 0.053	−0.030 ± 0.055	0.288	0.774	−2.196^a^	0.028*	3.078	0.003**
RPMSMA	0 ± 0.046	0 ± 0.047	−0.017 ± 0.063	−0.013	0.989	1.526	0.133	1.819	0.075
LM1	0.005 ± 0.054	−0.001 ± 0.048	−0.019 ± 0.065	−0.179^a^	0.858	2.065	0.044*	1.826	0.074
LS1	0.007 ± 0.042	0.001 ± 0.042	−0.009 ± 0.054	0.752	0.455	1.835	0.073	1.229	0.225
LPMSMA	−0.001 ± 0.044	−0.001 ± 0.043	−0.016 ± 0.054	−0.027	0.979	1.414	0.164	1.771	0.083

“L” represents the left side; “R” represents the right side. VRMS, virtual reality music stimulation; VRAO, virtual reality action observation; VRMI, virtual reality motor imagination; HBT, total hemoglobin; ROIs, brain regions of interest.

* 0.01 <* p* < 0.05.

** 0.001 < *p* < 0.01.

a Two related samples nonparametric test. And parametric *t* tests were used for the remaining comparative analyses.

In the homologous ROIs on HBO, VRMS exhibited a significant enhancement in LPMSMA compared with VRAO (*p* < 0.05) as determined by FDR (Fig. 3*c*; Table 3). For the homologous ROIs on HBT, VRMS demonstrated significant enhancement in RS1, LPMSMA, and RPMSMA compared with VRAO (*p* < 0.01) by FDR (Fig. 3*e*; Table 4). Furthermore, a significant enhancement was observed in LFPA (*p* < 0.05) by FDR under the same conditions (Fig. 3*e*; Table 4). Compared with VRMI, VRMS showed significant enhancement in RS1 (*p* < 0.05) by FDR (Fig. 3*e*; Table 4).

**Table 3.** Differences in FC for homologous ROIs between VRMS, VRAO, and VRMI in HBO signals

ROIs	Mean ± standard deviation			VRMS vs VRAO		VRMS vs VRMI		VRAO vs VRMI	
	VRMS	VRAO	VRMI	*t* value	*p* value (FDR)	*t* value	*p* value (FDR)	*t* value	*p* value (FDR)
LDLPFC	0.745 ± 0.191	0.782 ± 0.145	0.770 ± 0.186	−1.181	0.405	−0.748	0.619	0.443	1.099
RDLPFC	0.714 ± 0.222	0.632 ± 0.260	0.673 ± 0.278	2.095	0.103	1.199	0.590	−1.045	1.003
LS1	0.761 ± 0.152	0.747 ± 0.151	0.741 ± 0.153	0.654	0.737	0.975	0.619	0.293	1.101
RS1	0.806 ± 0.132	0.747 ± 0.183	0.743 ± 0.205	2.283	0.089	2.543	0.071	0.151	1.101
LPMSMA	0.740 ± 0.146	0.666 ± 0.179	0.681 ± 0.181	3.198	0.024*	2.725	0.071	−0.566	1.099
RPMSMA	0.736 ± 0.145	0.674 ± 0.176	0.689 ± 0.183	2.369	0.089	1.817	0.251	−0.548	1.099
LFPA	0.743 ± 0.151	0.677 ± 0.259	0.718 ± 0.242	1.854	0.139	0.846	0.619	−1.098	1.003
RFPA	0.760 ± 0.203	0.748 ± 0.184	0.780 ± 0.136	0.401	0.862	−0.688	0.619	−1.481	1.003

“L” represents the left side; “R” represents the right side. VRMS, virtual reality music stimulation; VRAO, virtual reality action observation; VRMI, virtual reality motor imagination; HBO, oxygenated hemoglobin; ROIs, brain regions of interest; FDR, false discovery rate; FC, functional connectivity; only one brain channel in brain regions LM1 and RM1 is not discussed.

* *p* < 0.05.

**Table 4.** Differences in FC for homologous ROIs between VRMS, VRAO, and VRMI in HBT signals

ROIs	Mean ± standard deviation			VRMS vs VRAO		VRMS vs VRMI		VRAO vs VRMI	
	VRMS	VRAO	VRMI	*t* value	*p* value (FDR)	*t* value	*p* value (FDR)	*t* value	*p* value (FDR)
LDLPFC	0.695 ± 0.269	0.751 ± 0.147	0.706 ± 0.252	−1.511	0.196	−0.281	0.975	1.482	0.483
RDLPFC	0.643 ± 0.263	0.534 ± 0.361	0.589 ± 0.359	2.095	0.083	1.136	0.436	−1.283	0.513
LS1	0.758 ± 0.146	0.722 ± 0.179	0.733 ± 0.167	1.536	0.196	1.185	0.436	−0.508	0.767
RS1	0.809 ± 0.121	0.722 ± 0.195	0.738 ± 0.221	4.023	0.002**	2.990	0.044*	−0.624	0.765
LPMSMA	0.717 ± 0.170	0.643 ± 0.213	0.680 ± 0.177	3.169	0.009**	1.674	0.335	−1.481	0.483
RPMSMA	0.702 ± 0.157	0.613 ± 0.219	0.653 ± 0.214	3.574	0.004**	1.808	0.335	−1.516	0.483
LFPA	0.653 ± 0.255	0.564 ± 0.353	0.604 ± 0.381	2.461	0.044*	1.263	0.436	−0.864	0.653
RFPA	0.704 ± 0.300	0.635 ± 0.352	0.672 ± 0.292	1.311	0.245	0.613	0.776	−1.135	0.524

“L” represents the left side; “R” represents the right side. VRMS, virtual reality music stimulation; VRAO, virtual reality action observation; VRMI, virtual reality motor imagination; HBT, total hemoglobin; ROIs, brain regions of interest; FDR, false discovery rate; FC, functional connectivity; Only one brain channel in brain regions LM1 and RM1 is not discussed.

* 0.01 <* p* < 0.05.

** 0.001 <* p* < 0.01.

**Figure 4.**
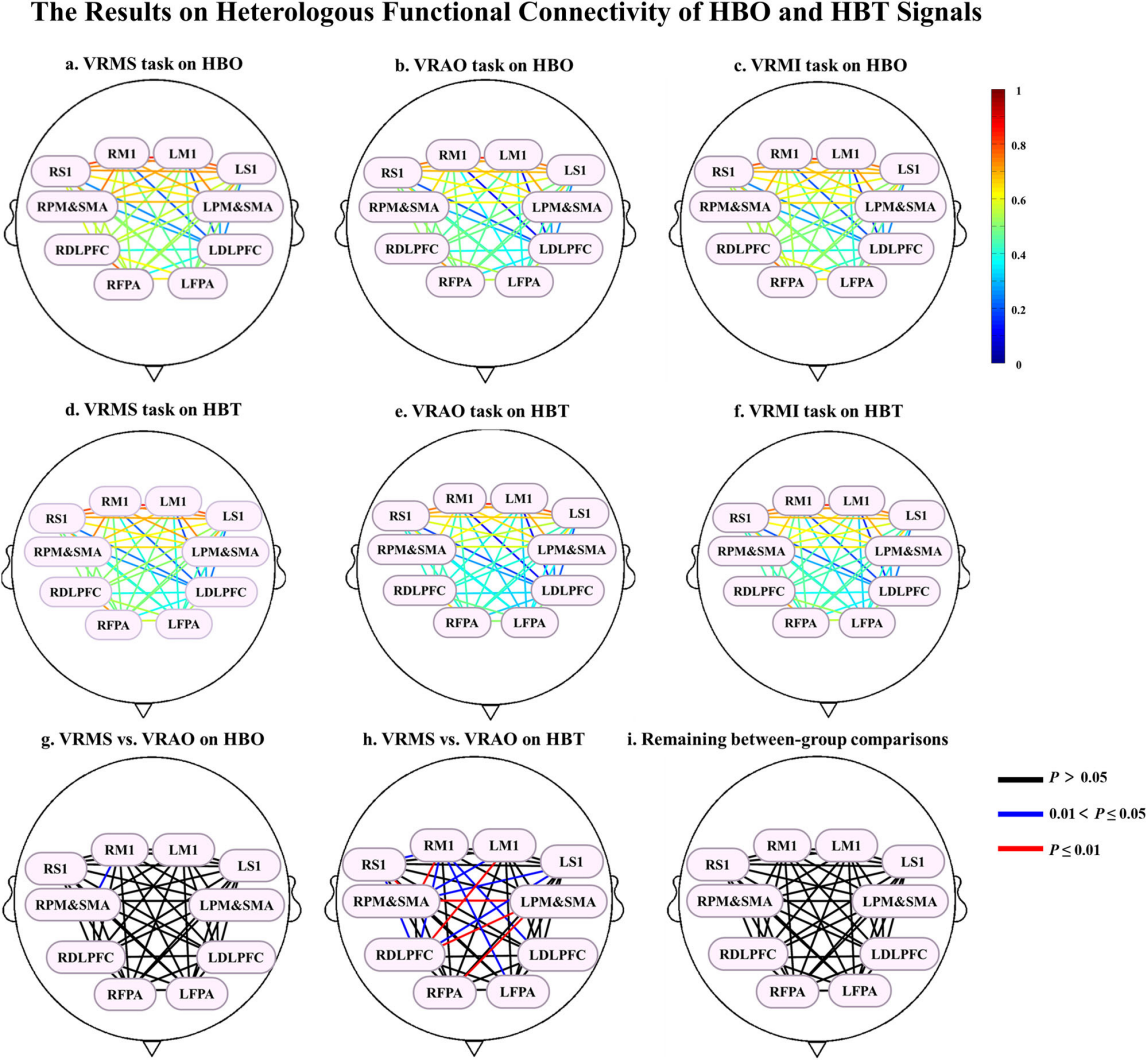
Heterogeneous brain-network connectivity results. VRMS, virtual reality music stimulation; VRAO, virtual reality action observation; VRMI, virtual reality motor imagination; HBO, oxygenated hemoglobin; ROIs, regions of interest. ***a–c***, Heterogeneous brain networks based on HBO for VRMS, VRAO, and VRMI, respectively. ***d–f***, Heterogeneous brain networks based on HBT for VRMS, VRAO, and VRMI, respectively. ***g***, ***h***, Heterogeneous brain networks based on HBT and HBO for the comparisons between VRMS and VRAO, respectively. ***i***, The remaining task comparisons showed no statistical significance. Significance levels: *p < 0.05; **p < 0.01. All the significant differences based on FDR correction.

In heterogeneous ROIs on hyperbaric oxygen therapy (HBO), the VRMS modality demonstrated significantly enhanced FC values in the RPMSMA and right primary motor cortex (RM1) compared with the visual and respiratory acoustic task open eyes (VRAO), as indicated by the FDR at *p* < 0.05 (Figs. 3*d*,*h*, 4*g*; Table 5).

**Table 5.** Differences in FC for heterologous ROIs between VRMS, VRAO, and VRMI in HBO signals

ROIs	Functional interaction brain regions	VRMS vs VRAO		VRMS vs VRMI		VRAO vs VRMI	
		*t* value	*p* value (FDR)	*t* value	*p* value (FDR)	*t* value	*p* value (FDR)
LDLPFC	RDLPFC	0.245	0.826	−0.278	0.929	−0.610	0.748
	LS1	0.256	0.826	0.137	0.942	−0.134	0.934
	RS1	1.366	0.236	0.221	0.929	−1.040	0.593
	LPM&SMA	0.552	0.640	0.047	0.963	−0.530	0.748
	RPM&SMA	−0.333	0.794	−0.448	0.929	−0.109	0.934
	LM1	1.283	0.250	0.882	0.748	−0.455	0.792
	RM1	1.967	0.099	−0.100	0.942	−2.071	0.410
	L FPA	−0.150	0.881	−0.763	0.809	−0.714	0.748
	RFPA	0.806	0.477	−0.630	0.913	−1.494	0.410
RDLPFC	LS1	2.760	0.061	1.608	0.474	−0.602	0.748
	RS1	2.137	0.089	1.482	0.474	−0.538	0.748
	LPM&SMA	2.553	0.069	1.680	0.474	−0.858	0.740
	RPM&SMA	1.547	0.193	1.647	0.474	−0.109	0.934
	LM1	2.790	0.061	0.147	0.942	−2.021	0.410
	RM1	2.912	0.061	0.905	0.748	−1.804	0.410
	LFPA	2.376	0.081	0.437	0.929	−1.895	0.410
	RFPA	2.151	0.089	0.289	0.929	−1.626	0.410
LS1	RS1	1.411	0.224	1.472	0.474	0.176	0.934
	LPM&SMA	1.878	0.115	2.092	0.474	0.175	0.934
	RPM&SMA	2.042	0.095	1.886	0.474	0.027	0.979
	LM1	0.902	0.429	1.063	0.694	0.535	0.748
	RM1	2.197	0.089	2.001	0.474	0.206	0.934
	LFPA	2.597	0.069	1.225	0.637	−1.058	0.593
	RFPA	1.984	0.099	0.416	0.929	−1.444	0.410
RS1	LPM&SMA	1.586	0.185	0.552	0.929	−1.138	0.586
	RPM&SMA	3.110	0.061	1.722	0.474	−1.076	0.593
	LM1	2.015	0.097	1.495	0.474	−0.568	0.748
	RM1	2.295	0.084	0.989	0.702	−1.508	0.410
	LFPA	2.376	0.081	0.605	0.913	−1.444	0.410
	RFPA	1.510	0.200	0.124	0.942	−1.281	0.516
LPM&SMA	RPM&SMA	2.212	0.089	1.713	0.474	−0.718	0.748
	LM1	1.332	0.243	1.081	0.694	−0.207	0.934
	RM1	1.793	0.132	1.029	0.694	−0.666	0.748
	LFPA	2.327	0.083	0.819	0.781	−1.463	0.410
	RFPA	2.764	0.061	1.062	0.694	−1.491	0.410
RPM&SMA	LM1	2.123	0.089	1.482	0.474	−0.670	0.748
	RM1	3.793	0.018*	1.856	0.474	−1.668	0.410
	LFPA	1.444	0.218	−0.339	0.929	−1.758	0.410
	RFPA	2.059	0.095	0.232	0.929	−1.558	0.410
LM1	RM1	1.310	0.245	1.682	0.474	0.535	0.748
	LFPA	2.114	0.089	1.228	0.637	−0.686	0.748
	RFPA	1.068	0.344	−0.272	0.929	−1.221	0.540
RM1	LFPA	2.667	0.066	0.486	0.929	−1.775	0.410
	RFPA	1.625	0.178	−0.373	0.929	−1.858	0.410
LFPA	RFPA	2.413	0.081	−0.309	0.929	−2.362	0.410

“L” represents the left side; “R” represents the right side. VRMS, virtual reality music stimulation; VRAO, virtual reality action observation; VRMI, virtual reality motor imagination; HBO, oxygenated hemoglobin; ROIs, brain regions of interest.; FDR, false discovery rate; FC, functional connectivity.

* *p* < 0.05.

In the heterogeneous ROIs on hyperbaric therapy (HBT), VRMS also showed significantly enhanced FC compared with VRAO in multiple regions: the LDLPFC and RM1, RDLPFC and left S1 cortex (LS1), RDLPFC and right S1 cortex (RS1), RDLPFC and RM1, LS1 and RPMSMA, RS1 and RM1, RPMSMA and left M1 cortex (LM1), and RM1 and LFPA, all at *p* < 0.05 by FDR (Figs. 3*f*,*j*, 4*h*; Table 6). Additionally, significant enhancements were noted for the RDLPFC and left PMA (LPMSMA), RDLPFC and LM1, RS1 and RPMSMA, LPMSMA and RPMSMA, LPMSMA and right FPA (RFPA), and RPMSMA and RM1 at *p* < 0.01 by FDR (Fig. 4*h*; Table 6).

**Table 6.** Differences in FC for heterologous ROIs between VRMS, VRAO, and VRMI in HBT signals

ROIs	Functional interaction brain regions	VRMS vs VRAO		VRMS vs VRMI		VRAO vs VRMI	
		*t* value	*p* value (FDR)	*t* value	*p* value (FDR)	*t* value	*p* value (FDR)
LDLPFC	RDLPFC	0.822	0.435	0.063	0.950	−0.874	0.689
	LS1	0.935	0.380	−0.106	0.950	−0.943	0.685
	RS1	1.483	0.197	0.567	0.737	−0.635	0.792
	LPM&SMA	1.615	0.159	0.075	0.950	−1.639	0.675
	RPM&SMA	0.480	0.641	−0.269	0.911	−0.656	0.792
	LM1	1.827	0.111	0.835	0.573	−0.852	0.689
	RM1	2.794	0.029*	0.743	0.629	−1.741	0.675
	L FPA	1.297	0.250	0.097	0.950	−1.322	0.681
	RFPA	0.997	0.355	−0.194	0.929	−1.089	0.681
RDLPFC	LS1	3.231	0.013*	2.501	0.252	−0.322	0.897
	RS1	3.041	0.019*	2.069	0.252	−0.997	0.681
	LPM&SMA	3.886	0.004**	2.175	0.252	−1.921	0.675
	RPM&SMA	2.131	0.081	1.860	0.254	−0.310	0.897
	LM1	3.819	0.004**	1.957	0.252	−1.649	0.675
	RM1	2.758	0.029*	1.300	0.409	−1.193	0.681
	LFPA	2.174	0.078	0.944	0.562	−1.196	0.681
	RFPA	2.115	0.081	0.395	0.822	−1.724	0.675
LS1	RS1	1.898	0.099	0.904	0.573	−0.985	0.681
	LPM&SMA	1.750	0.125	1.146	0.463	−0.668	0.792
	RPM&SMA	2.746	0.029*	1.441	0.390	−1.068	0.681
	LM1	0.469	0.641	1.233	0.419	1.013	0.681
	RM1	1.236	0.259	2.076	0.252	0.907	0.689
	LFPA	1.423	0.207	1.095	0.482	−0.249	0.928
	RFPA	2.083	0.083	1.404	0.395	−0.424	0.897
RS1	LPM&SMA	1.986	0.095	0.472	0.798	−1.429	0.681
	RPM&SMA	3.858	0.004**	1.886	0.254	−1.572	0.675
	LM1	1.915	0.099	1.986	0.252	−0.105	0.940
	RM1	2.857	0.028*	2.488	0.252	−0.165	0.940
	LFPA	1.231	0.259	1.308	0.409	0.382	0.897
	RFPA	2.284	0.071	1.493	0.376	−0.572	0.828
LPM&SMA	RPM&SMA	3.423	0.010**	0.952	0.562	−1.958	0.675
	LM1	1.282	0.250	1.308	0.409	0.318	0.897
	RM1	1.913	0.099	1.611	0.320	0.023	0.982
	LFPA	2.239	0.074	0.841	0.573	−1.519	0.675
	RFPA	3.420	0.010**	1.270	0.411	−2.115	0.675
RPM&SMA	LM1	3.205	0.013*	1.822	0.254	−0.129	0.940
	RM1	4.050	0.004**	2.440	0.252	−0.706	0.792
	LFPA	1.003	0.355	−0.224	0.926	−1.265	0.681
	RFPA	2.359	0.063	0.862	0.573	−1.175	0.681
LM1	RM1	2.016	0.092	2.069	0.252	−0.154	0.940
	LFPA	2.173	0.078	2.018	0.252	0.102	0.940
	RFPA	2.367	0.063	1.754	0.257	−0.352	0.897
RM1	LFPA	2.590	0.041*	1.793	0.254	−0.481	0.890
	RFPA	1.455	0.201	0.398	0.822	−0.978	0.681
LFPA	RFPA	1.959	0.097	0.641	0.694	−1.284	0.681

“L” represents the left side; “R” represents the right side. VRMS, virtual reality music stimulation; VRAO, virtual reality action observation; VRMI, virtual reality motor imagination; HBO, oxygenated hemoglobin; ROIs, brain regions of interest; FDR, false discovery rate; FC, functional connectivity.

* 0.01 < *p* < 0.05.

** 0.001 <* p* < 0.01.

## Discussion

This study examined the variations in brain activation and FC networks during MS, AO, and MI tasks within a VR context. The objective was to investigate how VR integration influences cerebral cortex hemodynamics by synchronizing the fNIRS during three distinct brain-stimulation tasks. Unlike AO and MI, MS had unique effects on the brain due to various elements of music theory, including tone, melody, rhythm, and music perception (Patterson et al., 2002). In research on event-related potentials, Palmer et al. (2009) found that modifying musical elements such as timbre, stress, and rhythm could alter the latency of mismatch negativity in listeners. This finding suggests that musical elements can regulate brain processing.

We observed similar effects of music on cortical hemodynamics using fNIRS monitoring. The comparisons indicated that the stimulating effects of VRMS on the brain's functional network connectivity were more pronounced than those observed with the other two tasks. Specifically, the VRMS results exhibited stronger functional connections in bilateral, heterogeneous, and homologous brain networks, particularly in the left SMA and PM regions. During VRMS stimulation, there was an enhancement in the functional connections of the bilateral PM and SMA regions with the RM1, RS1, RDLPFC, and bilateral FPA regions. However, the enhancement observed with VRMS was evident only in the HBT signal and not in HBO, potentially due to factors such as stability, effect size, or sample size.

This indicates that VRMS enhances the frequency of functional interactions among different brain regions and improves the efficiency of FC within these areas. Notably, the PM and the SMA are closely linked to both movement and beat perception and prediction (Cannon and Patel, 2021; Guida et al., 2023). This association motivated the investigation of the specific impact of musical stimulation on the PM and SMA.

### Specific effects of MS on the functional networks of motor and cognitive-related cortical regions

In the nonmotor state, MS facilitates the functional interaction between motor and cognitive-related brain areas. MS can induce rhythm perception in the brain, a complex activity requiring the collaboration of the auditory, cognitive, and motor cortices. According to Merchant et al. (2015) study, beat perception is based on the motor cortico-basal-ganglia-thalamo-cortical circuit, which transmits information via delta and beta oscillatory activities. During beat perception, the relevant effectors in motion-related areas activate simultaneously, enabling interaction between auditory and motion-related brain regions.

According to the auditory perception-induced action simulation for auditory prediction theory, brain regions responsible for movement are activated during beat perception, specifically the SMA and dorsal striatum (Cannon and Patel, 2021). The term “groove” describes the sensation of movement elicited by music. Subsequent studies have shown that predictive coding also applies to the phenomenon of “groove” (Vuust et al., 2022; Chen et al., 2025). This predictive coding helps minimize errors during “groove”-related activities. Matthews et al. suggested that motor timing and reward elements associated with the groove experience are linked to specific cortex-striatal pathways. When exposed to music or rhythm, the putamen, SMA, and PM develop internal representations of the beat that interact with the caudate as well as prefrontal and parietal regions (Matthews et al., 2020). Furthermore, Toiviainen et al. found that the functional connection strength and interaction patterns of SMA and PM-related brain networks can be influenced by varying beat levels. Low-beat musical stimulation increased the coupling response between the SMA and PM, potentially relating to movement occurrence or its prediction. In contrast, high-tempo stimulation strengthened connectivity between the SMA and the cerebellum, causing a shift in main network connections from the motor cortex to the cerebellum. Thus, previous studies have indicated that the SMA is a crucial brain region for the functional coupling of auditory and sensorimotor regions during rhythm and beat perception (Toiviainen et al., 2020).

In this study, the FC between the SMA and associated brain regions was enhanced under VRMS. This enhancement was particularly notable in regions related to cognition, motor function, and sensation in the right hemisphere (RM1, RS1, and RDLPFC). These findings suggest a coupling response of multiple brain areas, indicating a tendency toward right-brain lateralization during processing. Grossbach et al. similarly observed that bilateral frontal and temporal brain regions exhibited sustained cortical activation during the processing of musical rhythm and beat, with a right-brain processing advantage. The right hemisphere is generally considered the primary region involved in music processing. Furthermore, the auditory, cognitive, and motor-related regions of the right hemisphere show certain processing advantages during rhythm and beat processing (Kuck et al., 2003).

This study's results, when combined with previous research, suggest that VRMS may induce brain-network interactions with a right-lateralized processing tendency. This primarily occurs through enhanced FC in SMA-related brain regions. This pattern differs from the brain-network configuration stimulated by motor paradigms such as AO and MI and aligns more closely with the characteristic changes induced by music. These findings may offer a theoretical foundation for the clinical application of VRMS in treatment.

### Advantages and prospects of VR technology

VR is a 3D computer-generated simulation that allows individuals to interact with a virtual environment in a seemingly realistic manner through electronic devices (Emmelkamp and Meyerbröker, 2021). Immersive VR provides users with a subjective sense of immersion. Its high flexibility enables integration with various conventional cognitive and behavioral therapy methods, fostering the development of innovative treatment strategies and offering broad application prospects (Bruno et al., 2022). VR technology has the potential to induce neuroplasticity through several specific mechanisms. These mechanisms include enhancing interhemispheric balance, strengthening cortical functional connections, and increasing the mapping between muscles and the cerebral cortex of an affected limb. Additionally, VR can simultaneously improve neuroplasticity and related behavioral indicators, activate frontal cortex regions, and potentially involve mirror neurons (Hao et al., 2022).

VR has gained popularity as a technology in recent years. Whether used as a primary treatment modality or as an assistive application, VR offers excellent flexibility and controllability. It can be seamlessly integrated with conventional rehabilitation therapy to address limitations of traditional treatments and enhance therapeutic outcomes. For example, combining VR with AO, MI, or mental simulation techniques can augment the original therapeutic effects (Choi et al., 2020).

The combination of VR and AO effectively enhances functional integration between mirror neurons and the sensorimotor cortex, as well as the activity of associated neurons (Fan and Luo, 2022; Lakshminarayanan et al., 2023). VR, when paired with walking tasks, offers diverse scenarios for gait training in individuals with hemiplegia following a stroke (Chen et al., 2016). Additionally, the integration of VR and imagery therapy can significantly improve upper-limb function (Jo et al., 2024). Moreover, VR can be incorporated into exposure therapy to simulate situations such as highly stressful scenes and social interactions, benefiting individuals with anxiety disorders, post-traumatic stress disorder, and other psychological issues (Natali et al., 2024).

In summary, integrating VR technology has the potential to revitalize traditional rehabilitation methods by fostering a more diverse and adaptable approach. This integration might decrease labor costs for hospitals and rehabilitation institutions while addressing the limitations inherent in standard rehabilitation practices. Additionally, it could overcome site constraints, offer economic advantages, and ultimately contribute to technological advancement (Stamm et al., 2020).

### Limitations

A limitation of this study was its inclusion of only young participants aged 18–22 years, which resulted in an insufficient sample size and age stratification. The fNIRS data collection process is vulnerable to various factors, including environmental lighting, the subjects’ skull thickness, and gender, which can cause unstable reception of the light source. Moreover, the fNIRS technique is prone to optical interference from hair and scalp, introducing uncertainty into the experimental data. Additionally, due to the delay in hemodynamic response, the time resolution of fNIRS is low, complicating the capture of the entire task effect.

This research necessitated complex data processing and analysis, including noise removal and data correction, which increased the complexity and duration of data processing, as well as the uncertainty of experimental results. Our study was limited by the 24 channels of fNIRS, which can only cover the cognitive and motor brain regions, without the ability to simultaneously monitor the visual or auditory ROIs. Consequently, further discussion on the effect of integrating VR with conventional rehabilitation technology on brain stimulation was not possible. Future studies should consider using multichannel fNIRS that covers the entire brain region to investigate VR's effect comprehensively.

Moreover, the task sequence in our experiment could introduce temporal sequence-induced fixed effects, posing another limitation. The order of tasks could influence experimental outcomes due to neural adaptation and resource depletion mechanisms. Particular attention should be given to cumulative neuroplastic effects, especially in VRAO and VRMI paradigms. In our study, HBO and HBT signals served as primary data, demonstrating rapid baseline recovery (5–7 s) under nonstimulated states. When integrated with a block-averaging paradigm methodology, these design features could reduce temporal sequence-induced fixed effects.

## Conclusion

VR combined with conventional rehabilitation represents an innovative approach in neural stimulation. The findings suggest that the stimulating effects of VRMS may enhance brain networks, particularly in PM and SMA-related regions. These results highlight the potential of VR technology for future clinical applications. Future research should explore the efficacy of integrating VR with conventional rehabilitation therapies to advance both VR technology exploration and its clinical implementation.

## Data Availability

All data included in this study are available upon request by contact with the corresponding author.

## Synthesis

Reviewing Editor: James Howard, Brandeis University Decisions are customarily a result of the Reviewing Editor and the peer reviewers coming together and discussing their recommendations until a consensus is reached. When revisions are invited, a fact-based synthesis statement explaining their decision and outlining what is needed to prepare a revision will be listed below. The following reviewer(s) agreed to reveal their identity: Jingwen Li, Jung Uk Kang.

We thank you for your response to the suggestions, including clarification regarding the statistical methods, analyses, and subject characteristics, and using rank-sum test instead of signed-rank test for non-normally distributed data.
